# Crystallization Kinetics of Modified Nanocellulose/Monomer Casting Nylon Composites

**DOI:** 10.3390/polym15030719

**Published:** 2023-01-31

**Authors:** Xiaofeng He, Fuqiang Guo, Kaihong Tang, Tiejun Ge

**Affiliations:** 1 Department of Polymer Science and Engineering, Shenyang University of Chemical Technology, Shenyang 110142, China; 2 Liaoning Polymer Materials Engineering and Technology Research Center, Shenyang 110142, China

**Keywords:** nanocellulose, MC nylon, crystallization kinetics, activation energy

## Abstract

Polyisocyanate and caprolactone were used to chemically functionalize nanocellulose (CNF). Composites of CNF, caprolactone-modified nanocellulose (CNF–CL) and polyisocyanate-modified nanocellulose (CNF–JQ)/MC nylon were fabricated by anionic ring-opening polymerization. The effects of the crystal structure, crystal morphology and crystallization process of MC nylon composites have been characterized by wide-angle X-ray diffraction (WAXD), polarized optical microscopy(POM) and differential scanning calorimetry (DSC). Isothermal crystallization kinetics were analyzed using the Avrami equation, and the crystallization rate, half-time, and Avrami exponent were calculated. The results show that the nucleation effects of CNF–JQ/MC nylon composites is increased with the CNF–JQ increase, and it is best compared with MC nylon, CNF/MC nylon and CNF–CL/MC nylon composites, so CNF–JQ can play the role of effective nucleating agent in MC nylon. We also discussed the non-isothermal crystallization of the composites. Analysis of the Jeziorny and Mo model demonstrates that the Zc values of CNF, CNF–CL, CNF–JQ/MC nylon composites increase, and the F(T) values decrease in order. This indicates that CNF–JQ can better promote the crystallization rate of non-isothermal crystallization of MC nylon. The results of this work demonstrate that CNF–JQ can be an effective nucleation agent and increase the crystallization rate of MC nylon compared with CNF–CL. The activation energy of the composites was studied using the kissing method, and the results showed that CNF–CL decreased the activation energy of MC nylon, and CNF and CNF–JQ increased the activation energy of MC nylon.

## 1. Introduction

Monomer Casting Nylons, called MC nylons, are important engineering plastics. It is widely used because of its excellent properties, including high mechanical and corrosion resistance, self-lubricating properties and modulus, as well as wear resistance and good molding and processing abilities. Among the polyamide nylons, MC nylon is not only the representative and most common, but also the most popular semi-crystalline engineering plastic because of its excellent mechanical properties, self-lubricity, high strength and good corrosion resistance [1]. The crystallization of MC nylon polymers ultimately affects their properties. Many scholars have studied the crystallization mechanism of MC nylon, and it is still the subject of many scientific studies [2]. MC nylon is polymerized while crystallizing and crystallizes rapidly. As a result, product shrinkage and dimensional instability can occur, which can adversely affect the performance of the polymer and its use. Therefore, it is important to study the crystallinity and crystallization parameters of MC nylon. The Avrami equation is the classical theory for the study of polymer crystallization kinetics; however, it was originally developed for isothermal crystallization processes and cannot be applied to non-isothermal processes that are more closely related to the actual molding of composites, so a lot of research has been conducted to modify the Avrami equation [3]. Nakamura and Ziabicki with Jarecki extended the Avrami equation to calculate non-isothermal crystallization kinetic parameters using isothermal crystallization data, and Ozawa extended the Avrami equation to non-isothermal processes with isothermal cooling by considering the effect of cooling rate. By correlating Avrami’s equation and Ozawa’s equation, Mo obtained a new crystallization kinetic model, which overcomes the shortcomings of each of the above two methods and achieves better applications in the study of non-isothermal crystallization kinetics of various polymers. Isothermal crystallization is the process of crystallization of a polymer at a constant temperature and is often described by the Avrami equation, which is a common experimental method used to characterize the crystallization behavior of polymers. Non-isothermal crystallization refers to the process of crystallization at different rates of temperature and is often described by Jeziorny’s method and Mo’s method. The current research on MC nylon is basically focused on synthesis, structural characterization, mechanical properties and thermal stability, and there are few systematic studies on crystallization behavior [4,5]. 

In recent decades, MC nylon composites with excellent properties have been extensively studied by many scholars. Nanofillers can make MC nylon produce good mechanical and thermal properties [6]. Nanofillers greatly influence the crystallization kinetics of MC nylon composites. The physical and mechanical properties of MC nylon composites depend not only on the dispersion and content of the filler, but are also closely related to the crystallization conditions. Previously, it has been shown that the filler as a nucleating agent can increase the rate and density of crystallization of MC nylon. For example, Yao Huimei et al. [7] analyzed the crystallization kinetics of PPES/MC nylon composites by Jeziorny model and found that primary and secondary crystallization mechanisms existed in all samples. The Zc values of in situ composites were lower than those of MC nylon, and the F(T) values of composites were generally higher than those of pure MC nylon. Moreover, the crystallization activation energy of in situ composites was lower than that of MC nylon, indicating that PPES can be used as an effective nucleating agent for MC nylon, and the movement of MC nylon chain segments is hindered during the nucleation process. MC nylon/SiO_2_ composites were prepared by an in situ polymerization method by Kim H B et al. [8]. The addition of SiO_2_ resulted in higher melting and crystallization temperatures and increased crystallinity. The crystallization induction time was reduced, which promoted the crystallization process of MC nylon and increased the crystal growth rate. Crystallization analysis showed that SiO_2_ particles acted as a crystallization promoter mainly by accelerating the nucleation of crystals. Qiu S C et al. [9] used toluene diisocyanate (TDI) to modify multi-walled carbon nanotubes containing hydroxyl groups and used the functionalized carbon nanotubes to prepare MC nylon/carbon nanotube composites. It was shown that carbon nanotubes acted as an effective nucleating agent in MC nylon, and the crystallization peak temperature of MC nylon increased and the crystallization temperature interval decreased, indicating that carbon nanotubes acted as a heterogeneous nucleating agent for MC nylon. The isothermal and non-isothermal crystallization kinetics of MC nylon and MC nylon/PAM composites were investigated by Xiongwei Qu by DSC. The Avrami equation was used to describe the isothermal crystallization stages in the composites, and the values of Avrami index obtained ranged from 1.70 to 3.28, indicating that different types of nucleation occurred simultaneously. The equilibrium melting point of MC nylon was enhanced with the addition of a small amount of PAM. A convenient method for analyzing the kinetics of non-isothermal crystallization of composites was obtained using the Mo method, a combination of Avrami and Ozawa equations [10].

Nanocellulose is nanoparticles up to a few micrometers in length, derived from wood and cotton, and is widely used for its remarkable properties and reproducibility. The high entanglement density allows it to demonstrate superior mechanical reinforcement potential in polymers. In addition, nanocellulose has the ability to improve thermal properties and gas barrier properties. Nanocellulose has a high specific surface area and volume, making it favorable for nucleation. As a heterogeneous nucleating agent, its dispersion in the polymer matrix is also important, and the compatibility of CNF with the matrix is even more so. Poor compatibility of CNF with the matrix leads to minimal aggregation and crystallization efficiency. The compatibility of nanocellulose with the substrate can be improved by various modifications. In our study, the modification of CNF uses hydroxyl groups present on the CNF surface, which can achieve uniform dispersion in MC nylon [11,12,13,14,15,16].

In many reports, MC nylon composites have been successfully prepared [17,18]; however, the effect of filler content on the properties of MC nylon composites has been thoroughly investigated, as well as on its crystallization kinetics, is lacking. In this paper, CNF was modified with isocyanate and caprolactone to improve the dispersion of CNF in the MC nylon. The crystallization kinetics of MC nylon was studied by differential scanning calorimetry, and the effects of the introduction of the third monomer on the crystallization behavior and melting behavior of MC nylon, as well as the effects of different modified fillers, filler content and crystallization conditions on the crystallization of MC nylon, were investigated. 

## 2. Materials and Methods

### 2.1. Materials

Industrial grade ε-caprolactam was purchased from Ube, Japan. Sodium hydroxide (NaOH 95%) was supplied by Maclin, which was analytically pure product and polyisocyanate as activator supplied by Hongshan Chemical Co., Ltd. (Liaoning, China). ε-caprolactone (ε-CL 97%), tin2-ethylhexanoate [Sn(Oct)_2_ 95%], and nanocellulose (CNF) were purchased from Shanghai Macklin Biochemical Co., Ltd. tetrahydrofuran (THF, p.a.), benzyl alcohol (BnOH 99%), acetone (99.8%), and methanol were purchased from DaMao Chemical Reagent Factory.

### 2.2. Sample Preparation

#### 2.2.1. Preparation of Polyisocyanate Modified CNF

**Figure 1:**
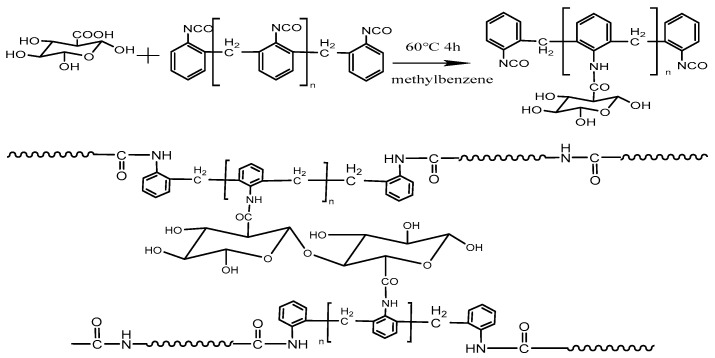
Preparation of CNF–JQ.

In the first step, 5 g CNF was added to toluene and dispersed well using ultrasound. In the second step, 15 g polyisocyanate was added to the solution drop by drop and the solution was stirred at 58 °C for 4 hours. Finally, the sample was washed with acetone to remove the unreacted polyisocyanate and dried, and then the modified CNF was obtained. Figure 1 shows the reaction mechanism of modified CNF(CNF-JQ).

#### 2.2.2. Preparation of ε-Caprolactam-Modified CNF

**Figure 2:**
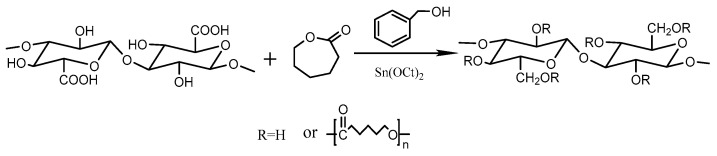
Preparation of CNF–CL.

First, 2 g of CNF and 40 g of ε-CL were uniformly dispersed in toluene using ultrasound. Then the initiator and catalyst Sn(Oct)2 were added, followed by three vacuums. The reaction was stirred at 110 °C for 24 h. It was precipitated in cold methanol. Finally, the soxhlet extraction was separated in THF at 80 °C and dried overnight. The modified CNF, named CNF–CL, was obtained, as shown in Figure 2.

#### 2.2.3. Synthesis of pure MC nylon

First, in order to melt the monomer, a three-neck flask containing 200 g CL was heated at 110 °C. The solution was refluxed under vacuum to remove the water from the solution. Next, 0.42 g NaOH was added to the solution as a catalyst and refluxed again under vacuum. After adding 7.06 g of polyisocyanate, the solution was stirred for 2 min and finally the melt was poured into the mold at 180 °C. As a result, MC nylon products were obtained.

#### 2.2.4. Preparation of Modified CNF/MC Nylon Composites

The preparation process of MC nylon/modified CNF composite is the same as that of pure MC nylon, except that the modified CNF can be added after the melting of CL.

### 2.3. Characterization

(1) Fourier Transform Infrared (FT-IR)

CNF, modified CNF and potassium bromide powder were blended and pressed into tablets, and the structure of the modified CNF was characterized using Fourier transform infrared spectrometer (Nicolet IS/10).

(2) Wide-angle X-ray diffraction (WAXD)

A tongda TD-300 WAXD (Dandong, China) was used to study the crystalline structures. The diffraction angle (2θ) of the scan is from 5° to 80° and the scan rate is 5° × min^−1^.

(3) Polarized optical microscopy (POM)

The spherical crystal morphology of pure MC nylon and MC nylon composites was observed by a polarized optical microscope (XP-201) equipped with an XPR-201 heating table. All samples were first melted above the melting point of MC nylon, and then crystallized at its crystallization temperature to observe the crystalline shape.

(4) Differential scanning calorimetry (DSC)

TA Instrument (Q-200) was used to studied the crystallization kinetics of the MC nylon composites. MC nylon composites were heated to 260 °C and then cooled to various crystallization temperatures (Tc) at a cooling rate of 100 °C × min^−1^ were used to study the isothermal crystallization kinetics. On the other hand, the specimens were maintained at 260 °C for 2 min and then cooled at different cooling rates. The specimens were made to crystallize under a non-isothermal cooling process to discuss the non-isothermal crystallization behavior of the composites..

## 3. Results and Discussion

### 3.1. Structural Analysis of Modified CNF

**Figure 3:**
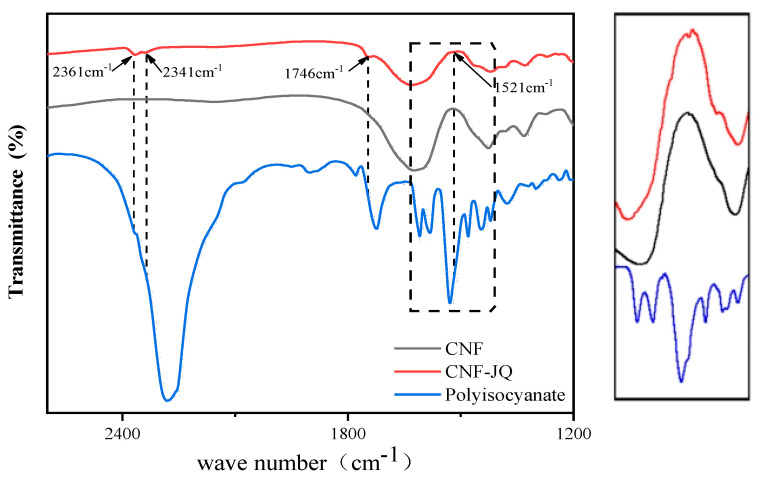
FTIR spectra of CNF, polyisocyanate, and CNF−JQ.

Figure 3 shows the FTIR spectra of CNF, polyisocyanate, and CNF–JQ. It can be observed that no absorption peak of -C=O- appears in the infrared spectrum of CNF. On the contrary, in the IR spectrum of CNF-JQ, the absorption peak at 1740 cm^−1^ appears to represent -C=O-. The peak at 1520 cm-1 indicates -NH, indicating the reaction of the isocyanate group with the carboxyl group. Peaks at 2341 cm^−1^ and 2361 cm^−1^ are absorption peaks of -N=C=O, indicating the presence of additional isocyanate groups as activators that can participate in the reaction of MC nylon [19]. 

**Figure 4:**
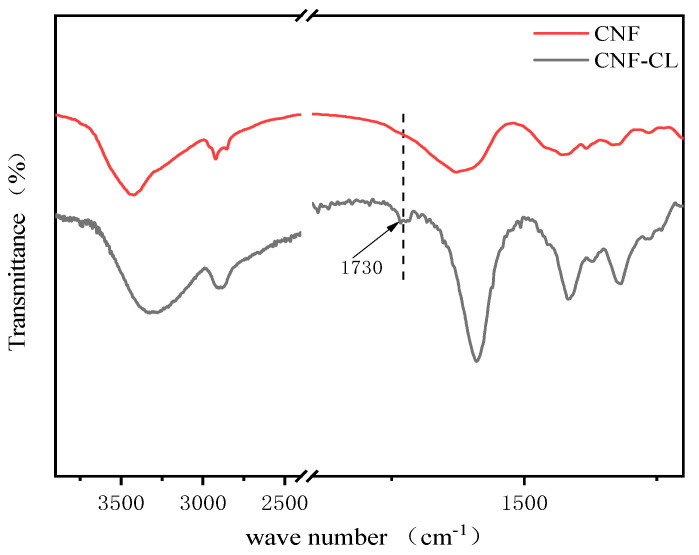
FTIR spectra of CNF and CNF−CL.

Figure 4 shows the FTIR spectra of CNF and CNF–CL. The FT-IR spectrum of CNF–CL showed a new characteristic peak at 1730 cm^−1^, which is the absorption peak of C=O in the grafted PCL ester group with stretching vibration, indicating the successful grafting of PCL onto cellulose [20]. 

### 3.2. Crystal Structure Analysis

**Figure 5:**
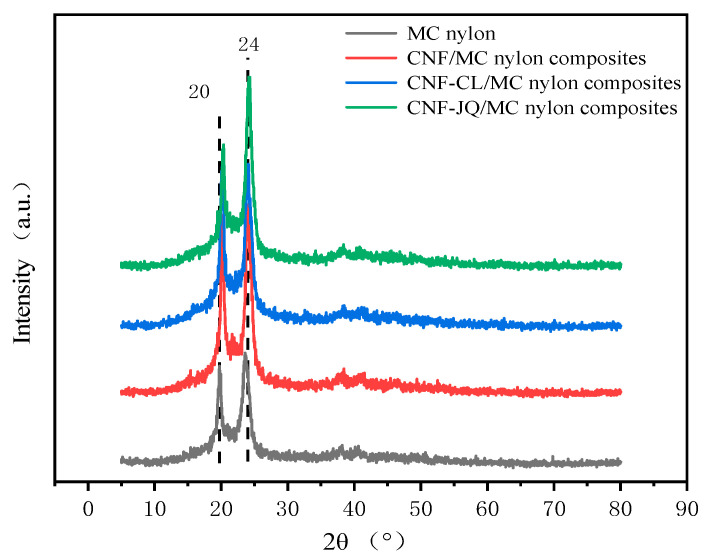
WAXD diffraction pattern of MC nylon and its composites.

Figure 5 shows the WAXD spectrum of the composite to analyze the effect of the modified CNF on the crystal structure of MC nylon. We can see that the WAXD curve include peaks at 20° and 24°, which are typical of MC nylon and correspond to the α-crystalline phase. Compared with MC nylon, there is no significant change in the range of diffraction peaks of MC nylon composites, indicating that they have the same crystalline structure [21]. 

### 3.3. Morphological Study of Crystallization

**Figure 6:**
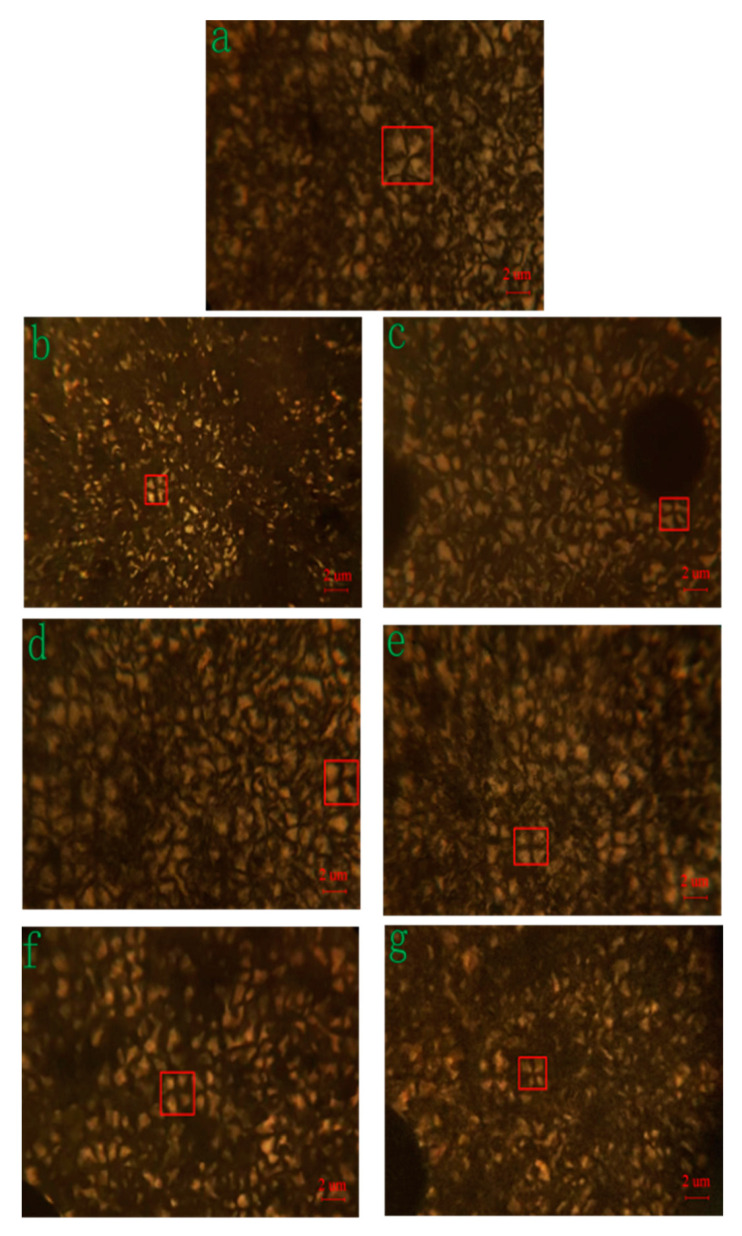
Polarized optical micrographs of MC nylon composites.

Figure 6 shows the crystalline morphology by polarized optical microscopy. Photomicrographs clearly reveal that the MC nylon has a larger, more developed spherulitic structure. In contrast, the crystal structure of composites is similar to MC nylon but much smaller than that. Because of heterogeneous nucleation for composites, the crystallites did not grow as large as MC nylon but increased nucleation density. In Table 1, the nucleation density of CNF, CNF–CL and CNF–JQ/MC nylon composites increased sequentially when the content of modified CNF was 1.0 wt%, indicating that the nucleation effect of CNF–JQ was better. The results of POM indicated that CNF–JQ was an effective nucleating agent for MC nylon. This is consistent with the conclusion from isothermal crystallization kinetics [22]. 

The quantities and sizes in the polarized micrographs calculated by Image-Pro Plus are shown in Table 1.

### 3.4. Isothermal Crystallization Kinetics

**Figure 7:**
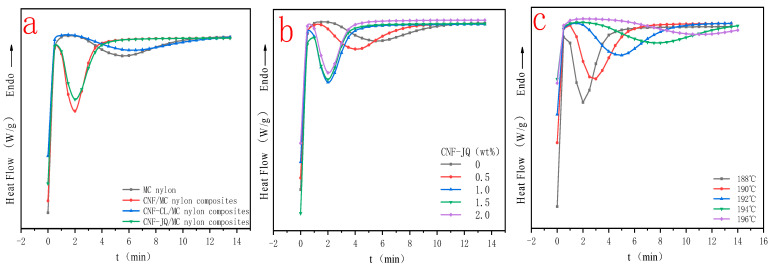
Heat flows as a function of time for MC nylon and MC nylon composites ((**a**) is the curve of different modified CNF with a filling of 1.0 wt% at a cooling rate of 188℃; (**b**) is the curve of different CNF–JQ additions at a cooling rate of 188 °C; (**c**) is the curve of different cooling rates for CNF−JQ of 1.0 wt%).

Figure 7a shows the isothermal crystallization curves of the composites with different modified CNF at the same temperature (188 °C); it can be seen that crystallization exothermic peaks and crystallization time of CNF–JQ/MC nylon become shorter. Still, the crystallization peaks of CNF–CL/MC nylon composites do not change much, indicating that the nucleation effects of CNF–JQ are better than CNF–CL. Therefore, the effects of different filler contents and crystallization temperatures on the crystallization kinetics are discussed using CNF–JQ/MC nylon composites as the matrix. Figure 7b shows the isothermal crystallization curves of the composites with different CNF–JQ contents at the same temperature (188 °C); it can be seen that the difference among the crystallization exothermic peaks of the composites with lower CNF–JQ content is not more significant than MC nylon. The peak shape and location of the exothermic peak were obviously different from those of MC nylon when the CNF–JQ content was higher. This indicates that the crystallization time of MC nylon was shortened more significantly with the increase of CNF–JQ content. Figure 7c shows the exothermic traces for the melt crystallization of semi-crystalline MC nylon at various isothermal crystallization temperatures. It is obvious from Figure 7c that as the crystallization temperature (Tc) increases, the crystallization exothermic peak becomes flat and shifts towards a longer time, which indicates that the sample takes longer to complete crystallization at higher crystallization temperatures. The increased nucleation-free energy at higher crystallization temperatures does not easily form nuclei and decreases the crystallization rate, and the isothermal crystallization behavior of MC nylon in situ composites strongly depends on the crystallization temperature [23,24]. 

The relative crystallinity of the polymers as a function of time enables a better understanding of the kinetics of isothermal crystallization of a series of MC nylon composites, which can be calculated from the enthalpy of heat generated during crystallization. In agreement with Equation (1) [25,26,27,28].

(1) $$X\left(t\right)=\frac{\int_{0}^{t}\frac{dH\left(t\right)}{dt}dt}{\int_{0}^{\infty }\frac{dH\left(t\right)}{dt}dt}=\frac{\Delta H_{t}}{\Delta H_{\infty }}$$

where dH(t)/dt is the exothermic rate; ΔH_t_ is the heat generated at moment t; and ΔH_∞_ is the total heat at the end of crystallization.

The crystallization kinetics of polymeric materials at isothermal conditions is usually analyzed by the Avrami equation, as follows [29,30].

(2) $$X_{t}=1-\exp\left(-{\mathrm{kt}}^{n}\right)$$

Taking the logarithm of both sides of Equation (2) yields Equation (3).

(3) lg[−ln(1−X(t))]=nlgt+lgk

where n is the Avrami index, which is related to the nucleation mechanism and characterizes the crystalline nucleation mode of the polymer, k and t are the isothermal crystallization rate constant and crystallization time, respectively.The isothermal crystallization kinetics of MC nylon composites were calculated from Equation (3). The parameters n and k are summarized in Table 2.

The half-crystallization time (t_1/2_) is the time required to reach 50% relative crystallinity and is one of the important variables in crystallization kinetics. The t_1/2_ can be calculated by Equation (4).

(4) $$t_{1/2}={\left(\ln2/K\right)}^{1/n}$$

Denote the crystallization rate by G, which is the reciprocal of the semi-crystallization time.

(5) $$G=1/t_{1/2}$$

**Figure 8:**
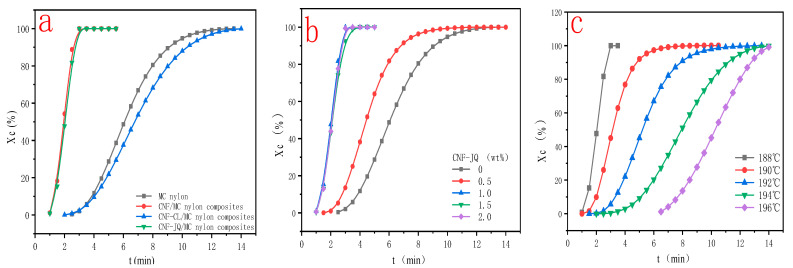
Relative crystallinity as a function of time for MC nylon and MC nylon composites ((**a**) is the curve for different modified CNF at a cooling rate of 188 °C and filler of 1.0 wt%; (**b**) is the curve for different CNF–JQ additions when cooling down to 188 °C; (**c**) is the curve for different temperatures when CNF–JQ is 1.0 wt%).

Figure 8 shows plots of X(t) versus crystallization time t for MC nylon and composites. From Figure 8a, the curves have the same sigmoid shape. The time required to complete of CNF–CL/MC nylon composites did not change much compared with pure MC nylon, but CNF–JQ and CNF/MC nylon composites were significantly shortened compared to the MC nylon. From Figure 8b,c, as the isothermal crystallization temperature decreases and the CNF-JQ content increases, the isothermal curve of the composite shifts to the left., indicating that the crystallization rates become faster. 

From Table 2, the *n* values of MC nylon and composites range from 2.962 to 4.225, indicating the crystallization mode might be a mixture with two-dimensional flakes and three-dimensional circular. Since the value of *n* is not an integer, the composite material constitutes both homogeneous and heterogeneous nucleation. The *n* value of the composite is lower than that of MC nylon because of the nucleation effect of modified CNF. There is a significant difference in the *n* value of crystallization temperature, n increases with increasing crystallization temperature. However, at higher crystallization temperatures, the exponent of MC nylon composites reaches 6.314. The results imply that MC nylon and MC nylon composites have the same nucleation and growth mechanism only at lower temperatures [31,32]. 

**Figure 9:**
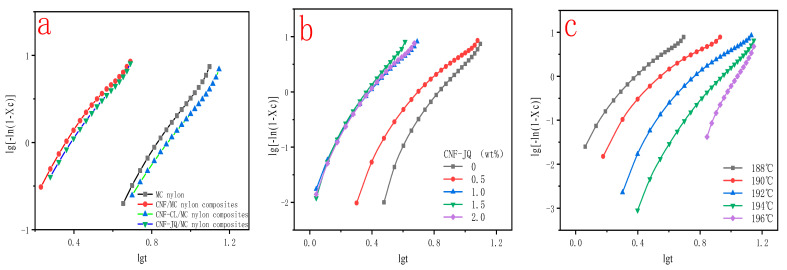
Avrami plots of MC nylon and MC nylon composites isothermally melt crystallized ((**a**) is the curve of different modified CNF with a filling of 1.0 wt% at a cooling rate of 188 °C; (**b**) is the curve of different CNF−JQ additions at a cooling rate of 188 °C; (**c**) is the curve of different cooling rates for CNF−JQ of 1.0 wt%).

**Figure 10:**
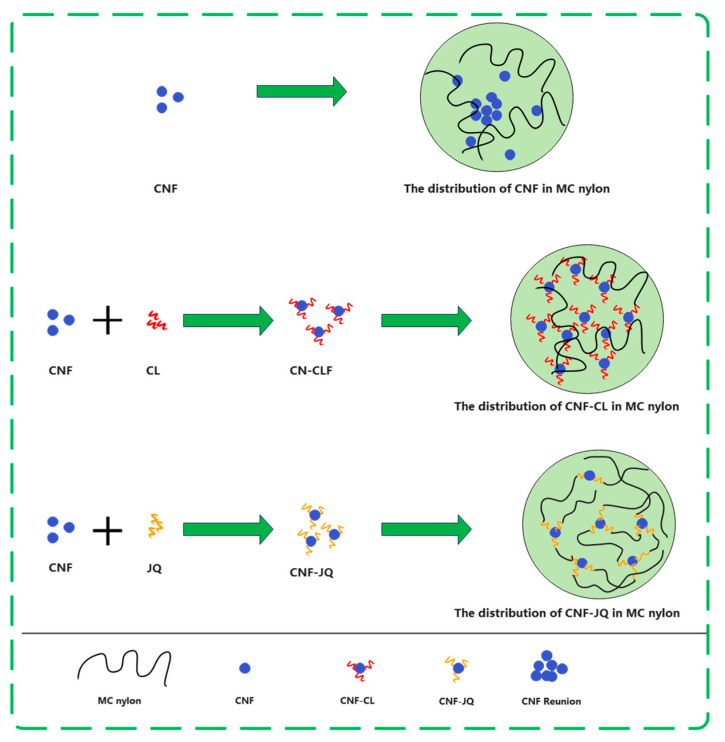
Dispersion mechanism of modified CNF in MC nylon matrix.

From Figure 9a and Table 2, the semi-crystallization time t_1/2_ of MC nylon composites is shorter than MC nylon, the values of G and k are larger than MC nylon. Because the modified CNF acts as a heterogeneous nucleation, the crystallization rate is increased. Crystallization rates is CNF–JQ > CNF > CNF–CL in order. To explain these phenomena, we propose the mechanism of adding CNF–CL and CNF–JQ dispersion in the MC nylon matrix, as shown in Figure 10, where the nucleation efficiency of the filler depends mainly on its concentration and distribution. When CNF is added to MC nylon matrix alone, it is difficult to achieve uniform distribution. The agglomeration of unmodified CNF in MC nylon will settle to the bottom of the mold, and the remaining small amount is well dispersed in MC nylon, which plays a better nucleation role than CNF–CL. The short chain of polycaprolactone in CNF–CL can be entangled with itself or MC nylon to improve its dispersibility. CNF–JQ can participate in the reaction of MC nylon by chemical reaction as a small molecule activator. CNF–JQ has the best dispersibility and the nucleation effect is more obvious. Therefore, the effects of different filler contents and crystallization temperatures on the crystallization kinetics are discussed using CNF–JQ/MC nylon composites as the matrix. From Figure 9b and Table 2, the k and G values of the CNF–JQ/MC nylon composites are higher than pure MC nylon. The value of t_1/2_ decreases when the amount of CNF–JQ is increased, when the CNF–JQ content is 1.0 wt%, the composite has the largest G value and the smallest t_1/2_. The present results again demonstrate that the addition of CNF-JQ can significantly improve the isothermal crystallization rate of MC nylon. CNF–JQ can be suggested as an efficient nucleation agent for the isothermal crystallization of MC nylon. From Table 2 and Figure 9c, the crystallization rate G and crystallization rate constant k value for CNF–JQ/MC nylon composites decreased and the semi-crystallization time t_1/2_ increased with the Tc. This is because of the difficulty of crystal nucleation at high temperature. The isothermal crystallization behavior of the composite is better at lower crystallization temperatures [33]. 

### 3.5. Non Isothermal Crystallization Kinetics

**Figure 11:**
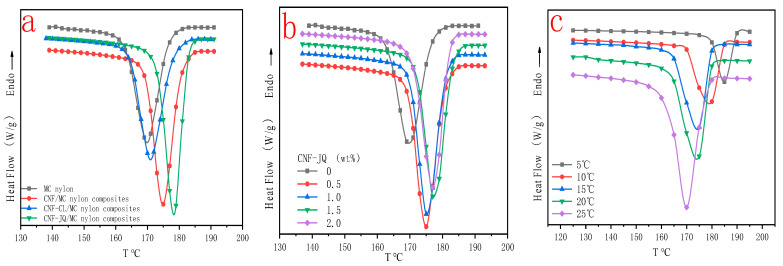
Temperature versus heat flow curves for MC nylon and its composites ((**a**) for different modified CNF at a cooling rate of 10 °C with 1.0 wt% filler; (**b**) for different CNF–JQ additions at a cooling rate of 10 °C; (**c**) for different cooling rate curves at 1.0 wt% CNF–JQ).

Figure 11a shows the DSC curves of different modified CNF/MC nylon composites with a cooling rate of 10 °C and filler content of 1.0 wt%. Compared with pure MC nylon, the crystallization temperatures of CNF, CNF–JQ and CNF–CL/MC nylon composites shifted toward higher temperatures, indicating that the addition of modified CNF can promote the crystallization of MC nylon. However, the crystallization temperatures of CNF and CNF–CL/MC nylon composites were lower than CNF–JQ/MC nylon composites, so CNF–JQ can better promote crystallization. Figure 11b shows the DSC curve of CNF–JQ/MC nylon composites with a cooling rate of 10 °C. The crystallization exothermic peak of the composites gradually becomes narrower, and the crystallization temperature shifts toward higher temperatures with the increase of CNF–JQ content. This indicates that CNF–JQ makes the heterogeneous nucleation of the composite more obvious with increasing content. Figure 11c shows the DSC curves of the specimens at different cooling rates; the crystallization exothermic peak shifts toward lower temperatures with the cooling rate increases. When MC nylon is crystallized at a lower cooling rate, the chain segments have enough time to flow as well as grow into microcrystals; however, when the cooling rate is faster, the chain segments of MC nylon are frozen before forming regular microcrystals, thus lowering the crystallization temperature [34,35,36,37]. 

**Figure 12:**
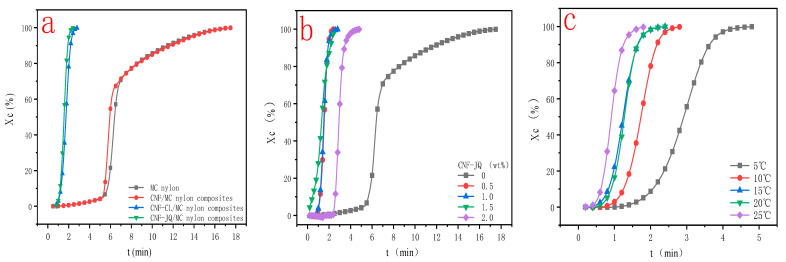
Curves of time versus crystallinity for MC nylon and its composites ((**a**) for different modified CNF at a cooling rate of 10 °C; (**b**) for different CNF–JQ additions at a cooling rate of 10 °C; (**c**) for different cooling rates at 1.0 wt% CNF–JQ).

The plots of the relative crystallinity versus temperature for MC nylon and modified CNF/MC nylon nanocomposite can be drawn through the integral of crystallization peak (Figure 12). It can be seen that all these curves have the same sigmoid shape. In Figure 12a, the time required to complete the crystallization of CNF/MC nylon composites did not change much compared with pure MC nylon, but CNF–JQ and CNF–CL/MC nylon composites were significantly shortened compared to MC nylon. As seen in Figure 12b, CNF–JQ/MC nylon composites completes the crystallization in less time than pure MC nylon at a given cooling rate, implying the addition of CNF accelerates the overall crystallization process. As can be seen from Figure 12c, the crystallinity curve becomes narrower and the crystallization completion time is shortened with the increase in cooling rate, and the rise of the curve slows down significantly in the late stage of crystallization, because with the increase of crystal size in the late stage, the crystals contact each other to form grain boundaries, which hinders and delays the continued growth of the crystals, and finally gradually tends to level off and stops crystallization.

#### 3.5.1. Jeziorny Method

The non-isothermal crystallization kinetics of polymer systems can be studied by the DSC method. Considering the characteristics of non-isothermal crystallization from the Avrami equation dealing with isothermal crystallization, some methods for dealing with non-isothermal crystallization kinetics can be derived by modifying the AvmIni equation. Non-isothermal crystallization data of pure MC nylon and composites are dealt according to the following equations [38]:

(6) $$1-X\left(t\right)=\exp\left(-Z_{t}\times t^{n}\right)$$

(7) $$\mathrm{lg}\left[-\ln\left(1-X\left(t\right)\right)\right]=lgZ_{t}+nlgt$$

where X(t) is the relative crystallinity at crystallization time t; Z_t_ is the crystallization rate constant, which is related to the crystallization temperature.

Plotting lgt against lg[−ln(1 − X(t))], the value of n can be obtained from the slope, while the value of Z_t_ can be obtained from the intercept. Taking into account the effect of the cooling rate and in order to make the equation more applicable to the analysis of non-isothermal crystallization behavior, a correction for Z_t_, which was given as follows:

(8) $${\mathrm{lgZ}}_{c}={\mathrm{lgZ}}_{t}/\phi$$

**Figure 13:**
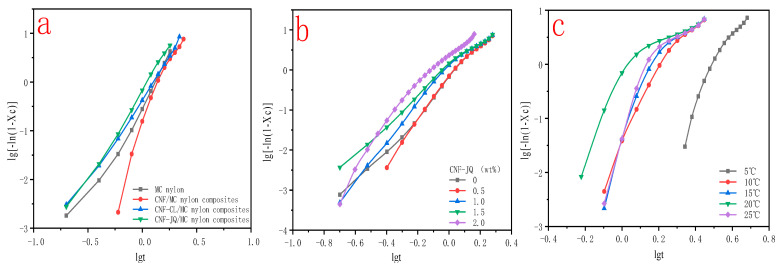
Curves of lgt versus lg[-ln(1-X(t))] for MC nylon and its composites ((**a**) for different modified CNF at 10 °C filler at 1.0 wt% cooling rate; (**b**) for different CNF−JQ additions at 15 °C cooling rate; (**c**) for different cooling rates at 1.0 wt% CNF−JQ).

The crystallization parameters of MC nylon and MC nylon composites were calculated using the Jeziorny method. It can be seen from Figure 13a and Table 3 that the Zc of MC nylon composites is higher than MC nylon, indicating that the modified CNF plays the role of heterogeneous nucleating agent, and the addition of modified CNF accelerates the crystallization rate of MC nylon. Modified CNF in MC nylon matrix can both reduce the free energy barrier of crystal nucleation and improve the activity of molecular chains, all of which can improve the orderliness of MC nylon molecular chain arrangement. Zc values of CNF, CNF–CL and CNF–JQ/MC nylon composites increase in order, this reason is explained by Figure 10, which indicates that CNF–JQ has the effect of better nucleation agent. From Figure 13b and Table 3, it can be seen that the Zc of the composites increased with the increase of CNF–JQ content. When the CNF–JQ content increases, the nucleation site and nucleation density of the composite increases (which is consistent with the POM results), and therefore the crystallization rate increases. It can be seen from Table 2 and Figure 13c, the value of Zc increases with the cooling rate increases, which indicates that the higher the cooling rate, the faster the crystallization rate of the composite. This is due to the fact that the free energy required for nucleation decreases as the cooling rate increases, leading to an increase in the crystallization rate [39,40]. 

#### 3.5.2. Mo Method

The new model combines the equations of Avrami and Ozawa found by Mo and coworkers to describe the non-isothermal crystallization behavior. The following relation was derived [41,42]:

(9) lgϕ=lgF(T)−αlgt

(10) $$F\left(T\right)={\left[K\left(T\right)/Z_{t}\right]}^{1/m}$$

where F(T) represents the cooling rate required to achieve a certain degree of crystallinity per unit of crystallization time; α = n/m is the ratio of Avrami and Ozawa indices.

**Figure 14:**
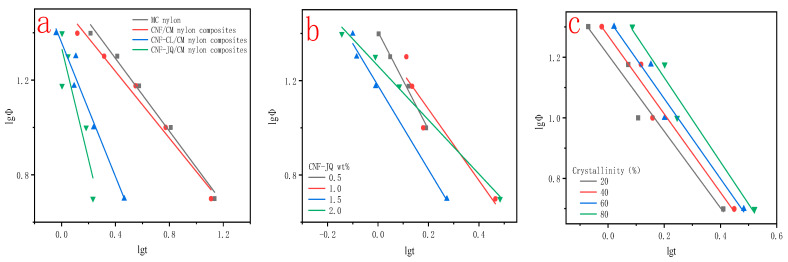
Curves of lgt vs. lgΦ for MC nylon and its composites ((**a**) for different modified CNF; (**b**) for different CNF−JQ additions; (**c**) for different crystallinity with CNF−JQ of 1.0 wt%).

Figure 14 shows the graph of lgΦ vs. lgt for MC nylon and its composites at a given crystallinity. It can be seen from the figure that lgΦ has a good linear relationship with lgt, which shows that the Mo method is reasonable to deal with the non-isothermal crystallization process in this experiment. The α and F(T) values are derived from the slope and intercept of the straight line in the figure, respectively, and are listed in Table 4.

From Figure 14a and Table 4 we can see that the F(T) values are CNF > CNF–CL > CNF–JQ in order. This result is consistent with the result of Jeziorny’s method. Figure 14b and Table 4 also show that the F(T) of MC nylon composites are smaller than MC nylon at the same relative crystallinity, which indicates that the addition of CNF–JQ can improve the crystallization rate of MC nylon and make crystallization relatively easy. When the content of modified CNF is 1.0 wt%, the crystallization of composites is the easiest. As shown in Table 4 and Figure 14c, it can be seen that the value of F(T) increase with increasing relative crystallinity. This is due to the fact that chain segments have a high degree of motility at low crystallinity, while at high crystallinity the motility of the chain segments is hindered by the previously formed crystal structure. Usually, a higher F(T) value means that more time is needed to reach a certain level of crystallinity. In other words, the higher the value of F(T), the slower the crystallization rate. These results are in agreement with those of the Jeziorny method. Those results are consistent with the result of Jeziorny’s method [43]. 

### 3.6. Activation Energy of Non-Isothermal Crystallization

Kissinger [44] proposed that the crystallization activation energy can be determined by the variation of the crystallization peak temperature with the cooling rate. The expression is as follows:

(11) $$\frac{d\left[\ln\left(\frac{\phi }{T_{p}^{2}}\right)\right]}{d\left(\frac{1}{T_{p}}\right)}=-\Delta \mathrm{Ea}/R$$

where Tp is the peak temperature, R is the gas constant and ∆Ea is the activation energy. 

The relationship between ln(Φ/T_p_^2^) and 1/Tp obtained from the Kissinger model. The crystallization activation energy (ΔEa) of the non-isothermal crystallization process can be calculated from the slope.

**Figure 15:**
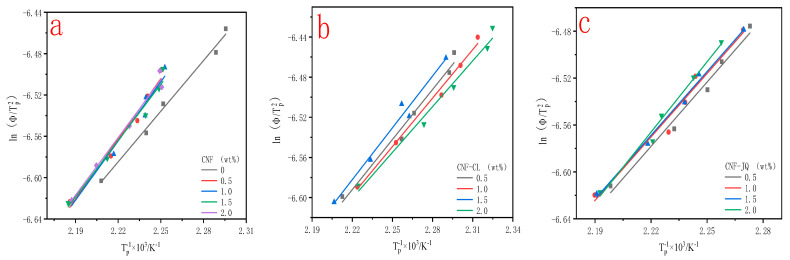
Curves of ln(Φ/T_p_^2^) versus 1/Tp for MC nylon and its composites ((**a**) is the activation curve of CNF; (**b**) is the activation curve of CNF−CL; (**c**) is the activation curve of CNF−JQ).

In the range of degrees of crystallinity, the ln(Φ/T_p_^2^) versus 1/T_p_ plot is shown in Figure 15. ΔEa can be obtained from the slope of the graph and is given in Table 5. We know that there are two different mechanisms by which CNF affects the crystallization process of polymers. One is that CNF can promote the crystallization of MC nylon by providing nucleation sites; the other is that CNF hinders the mobility of MC nylon chain segments. We find that the absolute value of ΔEa for CNF–CL/MC nylon is lower than that of pure MC nylon; this is because of the CNF–CL in composites acted as a heterogeneous agent to reduce the nucleation energy barrier. The absolute value of ΔEa for CNF and CNF–JQ/MC nylon is higher than that of pure MC nylon, indicating that the CNF and CNF–JQ in composites impede the movement of molecular chains to increase the nucleation energy barrier. In the composites, the heterogeneous nucleation is higher than the chain entanglement as the filler content increases and therefore the ΔEa value decreases [45]. 

## 4. Conclusions

In this work, the crystal structure, crystal morphology and crystallization kinetics of MC nylon and composites were investigated. WAXD analysis indicates that the peak intensity of the composites becomes larger than that of the neat MC nylon, but the structure of MC nylon does not change with variations in CNF content. The crystallite morphology of MC nylon, at different modified CNF content, was observed by polarizing optical microscopy. The nucleation density is improved and crystal size of MC nylon is reduced with the addition of modified CNF.

The isothermal crystallization results showed that the nucleation effect of CNF–JQ/MC nylon composite is more obvious compared with CNF and CNF–CL/MC nylon composites, which is related to CNF–JQ dispersion mechanism in MC nylon, and the rate constant k and crystallization rate G increase with increasing the content of CNF–JQ. That is, adding CNF–JQ in the composites can accelerate the crystallization. Non-isothermal crystallization behavior of MC composites analyzed by the Jeziorny and Mo model. The results showed that the Zc of composites increases from 0.899 to 0.962 and F(T) decreased with addition of different modified CNF, and the rate-dependent parameter of composites increased with the increase of CNF–JQ content. It demonstrates that the nucleation effect of CNF–JQ is better than CNF and CNF–CL, and nucleation effect becomes obvious with increasing content. The activation energy of the composites was studied using the kissing method, and the results showed that CNF–CL reduced the activation energy of MC nylon, and CNF and CNF–JQ increased the activation energy of MC nylon. 

Overall, by studying the Zc values in Jeziorny model, F(T) values in Mo model, and crystallization activation energy, it was concluded that CNF–JQ promoted MC nylon crystallization process and it is suggested as an efficient nucleation agent.

## Figures and Tables

**Table 1:** Nucleation density and grain size of MC nylon and MC nylon composites.

	MC Nylon (a)	CNF (wt%)		CNF–CL (wt%)		CNF–JQ (wt%)	
		0.5 (b)	1.0 (c)	0.5 (d)	1.0 (e)	0.5 (f)	1.0 (g)
**Number of Crystals**	452	554	511	463	594	620	626
**Diameter size (nm)**	3.78	2.22	2.35	2.95	2.50	2.33	2.19

**Table 2:** Kinetic parameters of isothermal crystallization of MC nylon and modified CNF/MC nylon composites.

		n	K (×10^−3^)	t_1/2_/min	G
Modified CNF	MC nylon	4.255	0.252	1.268	0.788
	CNF	2.962	8.312	0.432	2.313
	CNF–CL	3.023	2.572	0.648	1.543
	CNF–JQ	2.992	19.50	0.328	3.050
Content of CNF–JQ/wt% (cooling rate 188 °C)	0	4.255	0.252	1.268	0.788
	0.5	3.597	1.294	0.841	1.189
	1.0	2.996	19.50	0.328	3.046
	1.5	3.613	15.12	0.426	2.347
	2.0	3.104	15.53	0.367	2.723
Cooling rate/°C (CNF–JQ 1.0 wt%)	188	2.994	19.50	0.328	3.048
	190	3.192	11.75	0.412	2.427
	192	3.657	0.955	0.916	1.092
	194	4.625	0.039	1.863	0.537
	196	6.314	0.003	2.368	0.422

**Table 3:** Crystallization kinetic parameters from Jeziorny’s model.

		n	Z_t_	Zc
Modified CNF	MC nylon	3.628	0.344	0.899
	CNF	3.847	0.280	0.880
	CNF–CL	3.340	0.471	0.927
	CNF–JQ	3.587	0.679	0.962
Content of CNF–JQ/wt% (15 °C/min)	0	5.082	0.646	0.971
	0.5	5.534	0.692	0.976
	1.0	4.945	0.692	0.976
	1.5	3.786	0.741	0.980
	2.0	4.833	4.571	1.107
Φ (CNF–JQ 1.0 wt%)	5	6.412	0.481 × 10^−3^	0.217
	10	5.614	0.039	0.724
	15	4.945	0.692	0.976
	20	3.645	0.295	0.941
	25	5.557	0.054	0.890

**Table 4:** Parameters of crystallization kinetic based on Mo’s method.

		Relative Crystallinity (%)	F(T) (°C × min^−1^)
Modified CNF (1.0 wt%)	MC nylon	40	39.72
	CNF	40	33.04
	CNF–CL	40	22.86
	CNF–JQ	40	21.78
Content of CNF–JQ (wt%)	0	40	25.70
	0.5	40	21.78
	1.0	40	17.14
	1.5	40	18.20
	2.0	40	25.70
Content of CNF–JQ (wt%)	1.0	20	16.22
	1.0	40	17.14
	1.0	60	21.38
	1.0	80	25.70

**Table 5:** Non isothermal activation energy parameters of MC nylon and MC nylon composites.

		ΔEa (kJ/mol)
**MC nylon**		13.58
**Content of CNF (wt%)**	0.5	15.90
	1.0	15.12
**Content of CNF–CL (wt%)**	0.5	13.40
	1.0	12.65
**Content of CNF–JQ (wt%)**	0.5	15.16
	1.0	14.98

## Data Availability

Data available on request.
